# Association between city-wide lockdown and COVID-19 hospitalization rates in multigenerational households in New York City

**DOI:** 10.1371/journal.pone.0266127

**Published:** 2022-03-30

**Authors:** Arnab K. Ghosh, Sara Venkatraman, Evgeniya Reshetnyak, Mangala Rajan, Anjile An, John K. Chae, Mark A. Unruh, David Abramson, Charles DiMaggio, Nathaniel Hupert

**Affiliations:** 1 Department of Medicine, Weill Cornell Medical College, Cornell University, New York, New York, United States of America; 2 Department of Statistics and Data Science, Cornell University, Ithaca, New York, United States of America; 3 Department of Population Health Sciences, Weill Cornell Medical College, Cornell University, New York, New York, United States of America; 4 School of Global Public Health, New York University, New York, New York, United States of America; 5 Department of Surgery, New York University School of Medicine, New York, New York, United States of America; 6 Cornell Center for Disease and Disaster Preparedness, Weill Cornell Medical College, Cornell University, New York, New York, United States of America; UNICEF India, Government Medical College, INDIA

## Abstract

**Background:**

City-wide lockdowns and school closures have demonstrably impacted COVID-19 transmission. However, simulation studies have suggested an increased risk of COVID-19 related morbidity for older individuals inoculated by house-bound children. This study examines whether the March 2020 lockdown in New York City (NYC) was associated with higher COVID-19 hospitalization rates in neighborhoods with larger proportions of multigenerational households.

**Methods:**

We obtained daily age-segmented COVID-19 hospitalization counts in each of 166 ZIP code tabulation areas (ZCTAs) in NYC. Using Bayesian Poisson regression models that account for spatiotemporal dependencies between ZCTAs, as well as socioeconomic risk factors, we conducted a difference-in-differences study amongst ZCTA-level hospitalization rates from February 23 to May 2, 2020. We compared ZCTAs in the lowest quartile of multigenerational housing to other quartiles before and after the lockdown.

**Findings:**

Among individuals over 55 years, the lockdown was associated with higher COVID-19 hospitalization rates in ZCTAs with more multigenerational households. The greatest difference occurred three weeks after lockdown: Q2 vs. Q1: 54% increase (95% Bayesian credible intervals: 22–96%); Q3 vs. Q1: 48% (17–89%); Q4 vs. Q1: 66% (30–211%). After accounting for pandemic-related population shifts, a significant difference was observed only in Q4 ZCTAs: 37% (7–76%).

**Interpretation:**

By increasing house-bound mixing across older and younger age groups, city-wide lockdown mandates imposed during the growth of COVID-19 cases may have inadvertently, but transiently, contributed to increased transmission in multigenerational households.

## Introduction

Since COVID-19’s first appearance in December 2019, knowledge of transmission dynamics of SARS-CoV2 has rapidly advanced. Studies show that both proximity to infected patients and the concentration of inoculum play important roles in acquisition and subsequent severity of COVID-19 [1–5]. For this reason, public health measures that limit physical proximity (e.g. social distancing and lockdowns) have been critical to decreasing COVID-19 transmission and subsequent morbidity and mortality in the United States and abroad [6].

Evidence suggests that the organization of households and housing structure may also play a role in COVID-19 transmission. Ecological studies that examined links between housing conditions and COVID-19 rates describe associations between poorer housing conditions and COVID-19 cases [7], as well as between overcrowded housing and COVID-19 test positivity [8–11], even after accounting for numerous socioeconomic factors. Furthermore, multigenerational households may be at further risk because of the skewed nature of COVID-19 morbidity towards older individuals [12, 13]. In a nationwide study of multigenerational households in the United Kingdom during their first wave, men and women living in multigenerational households with children had a 17–21% increased risk of COVID-19 mortality after adjusting for several socioeconomic and clinical risk factors [14].

Non-pharmaceutical interventions (NPIs) such as city-wide lockdowns and school closures have had demonstrable impacts on COVID-19 transmission [15–17]. However, results from simulation modeling studies have suggested an increased risk of COVID-19 related morbidity after NPIs, including school closures. This risk is driven by increased inoculation of older individuals by house-bound school-age children [18], particularly in communities with limited means to reduce other potential exposure [19]. This hypothesis has not been empirically tested using real-world data. To explore this, we investigated whether the New York City (NYC)-wide lockdown in the spring of 2020 was associated with increased COVID-19 hospitalization rates in ZIP codes with higher proportions of multigenerational households. We hypothesized that after lockdown, COVID-19 hospitalization rates among older residents would be disproportionately higher in such ZIP codes, due to older individuals at high risk of contracting COVID-19 sharing the same indoor household spaces as asymptomatic school-aged children.

## Methods

### Study setting

**Fig 1:**
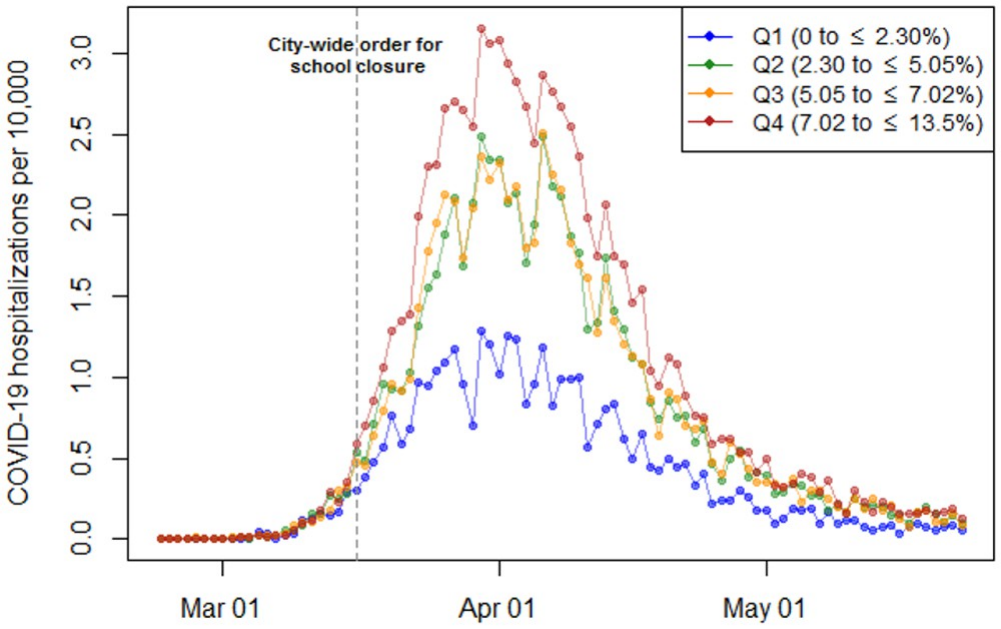
Unadjusted trends in COVID-19 hospitalizations per 10,000 in New York City (NYC) by quartiles of multigenerational households, February 23 to May 23, 2020.

We conducted a retrospective difference-in-difference analysis of weekly COVID-19 hospitalizations per 10,000 population in each NYC ZCTA between February 23 and May 23, 2020. Beginning in late February 2020, NYC became the global epicenter of the COVID-19 pandemic, with a first wave that crested in late March/early April 2020 (Fig 1). A city-mandated closure of all schools starting March 16, 2020 was the first public health measure to address the growing tide of COVID-19 cases [20], followed by a state-wide lockdown four days later [21].

### Variables and data sources

Our dependent variable was weekly COVID-19 hospitalization counts in each ZCTA, which were aggregated from daily hospitalization counts obtained from the NYC Department of Health and Mental Hygiene (NYC DOHMH). These counts were used to calculate weekly hospitalization rates per 10,000 population in each ZCTA, both across all patients and those over 55 years of age. We then calculated as our primary independent variable the proportion of households in each ZCTA that are multigenerational (defined as the percentage of households occupied by a grandparent and a grandchild less than 18 years of age), using the American Community Survey 5-year 2018 estimates. The primary analysis focused on hospitalized patients aged 55 years and older to reflect the average age of grandparents in the US [22]. Secondary analyses examined hospitalized patients of all ages.

Although several socioeconomic factors have been reported to be associated with increased rates of COVID-19 cases, we were careful to consider the potentially mediating effect of these factors in order to avoid over-adjusting our model of hospitalization rates over time and space. To account for socioeconomic conditions that likely confound the ecological-level association between the lockdown, COVID-19 hospitalization rates in ZCTAs, and multigenerational households, we modified the conceptual model used by Nalfilyan et al. [14]; this study described the relationship between household composition, socioeconomic status, and COVID-19 related risk.

Our primary analysis therefore included, by ZCTA, the proportion of residents who are White, the proportion of residents living under the federal poverty line (both as a percentage), and the median income (in 2018 US dollars). Furthermore, as we did in prior work on the first COVID-19 wave in NYC [23], we also included as a control covariate the ZCTA-level proportion of households that are overcrowded. This proportion was defined as the estimated number of housing units with more than one occupant per room, divided by the number of occupied housing units, expressed as a percentage. All measures of these covariates were taken from the American Community Survey (ACS) 5-year 2018 estimates.

In order to undertake inferential spatial analyses, spatial shapefiles of NYC’s ZCTAs were downloaded from the New York City Department of City Planning.

### Outcome

The primary outcome of interest was the adjusted COVID-19 weekly hospitalization rate per 10,000 population within each ZCTA for a) hospitalized patients over 55 years old, and b) for all hospitalized patients. We binned the ZCTA-level proportions of multigenerational households into quartiles and then calculated these hospitalization rates for each quartile, using the first (lowest) quartile as the reference group.

### Statistical analysis

Upon dividing NYC ZCTAs into quartiles of multigenerational housing percentages, we first compared the number of COVID-19 hospitalizations per 10,000 population in each quartile with the quartile’s socioeconomic characteristics (Table 1). Visual depictions of the hospitalization rates were created to describe the trajectory of the COVID-19 pandemic across the four quartiles.

**Table 1:** New York City (NYC) ZIP code tabulation area (ZCTA)-level COVID-19 hospitalization cases^1^, rates, and socioeconomic characteristics by proportion of multigenerational households in quartiles, February 23 to May 23, 2020.

	Proportion of Multigenerational Households^§^, in quartiles			
	First	Second	Third	Fourth
**Quartile cut-offs**	**0 to ⩽ 2.30%**	**2.30 to ⩽ 5.05%**	**5.05 to ⩽ 7.02%**	**7.02 to ⩽ 13.5%**
Total hospitalized COVID-19 cases	4760	11,867	14,907	19,474
Total hospitalized COVID-19 cases per 10,000	33.95	62.51	63.14	82.21
**ZCTA-level socioeconomic characteristics** ^2^				
Proportion of White residents, %	72.69	50.22	43.47	20.57
Proportion below federal poverty line, %	11.07	19.00	21.56	25.54
Median income, USD 2018^3^	105,963.90	63,411.35	55,243.78	49,601.94
Proportion of overcrowded households, ^¶^ %	4.92	7.99	9.59	13.54

^1^ Calculated from NYC Department of Health and Mental Hygiene COVID-19 data.

^2^ Derived from 2018 American Community Survey 5-year estimates.

^3^ Calculate using population-weighted average of the median incomes in the ZCTAs in each quartile.

^§^ Multi-generational households defined as the estimated number of residences occupied by grandparent and a grandchild less than 18 years of age.

^¶^ Overcrowded households defined as estimated number of housing units with more than one occupant per room, divided by the number of occupied housing units, expressed as a percentage.

Using a modified difference-in-difference analysis, we then estimated the association between the city-wide lockdown and hospitalization rates within each quartile of multigenerational household percentages. A typical difference-in-difference analysis defines an unexposed control group and an exposed treatment group; in our analysis, the exposure is the city-wide lockdown beginning with school closures on March 16, 2020. Our modified difference-in-difference analysis hypothesized an effect of increasing magnitude on COVID-19 hospitalization rates for ZCTAs with higher proportions of multigenerational households (quartiles 2–4) relative to quartile 1. Therefore, for the purposes of this analysis, we defined the control group as the ZCTAs in the first quartile of multigenerational households.

We could not test the parallel trend assumption of the difference-in-difference methodology directly. However, we assessed trends in the daily and weekly COVID-19 hospitalization rates by multigenerational housing quartile prior to the lockdown. Furthermore, we believe the parallel trend assumption holds as the decision for the lockdown was unlikely to be associated with other factors; it was abruptly implemented at the same time across all ZCTAs.

In our analysis, we estimated multivariable generalized linear models specifying a Poisson distribution for the weekly COVID-19 hospitalization counts. We included in these models a binary indicator that denoted whether each week under consideration occurred before or after the city-wide lockdown order. This allowed us to compare the COVID-19 hospitalization rates across the quartiles of multigenerational housing in weekly increments before and after the lockdown took effect. In light of the known spatial clustering of COVID-19 cases in NYC, we used the Moran’s I statistic to formally assess spatial autocorrelation of the cumulative COVID-19 hospitalizations rates by ZCTA over the study period. Details of our model specification are provided in the S1 File.

**Fig 2:**
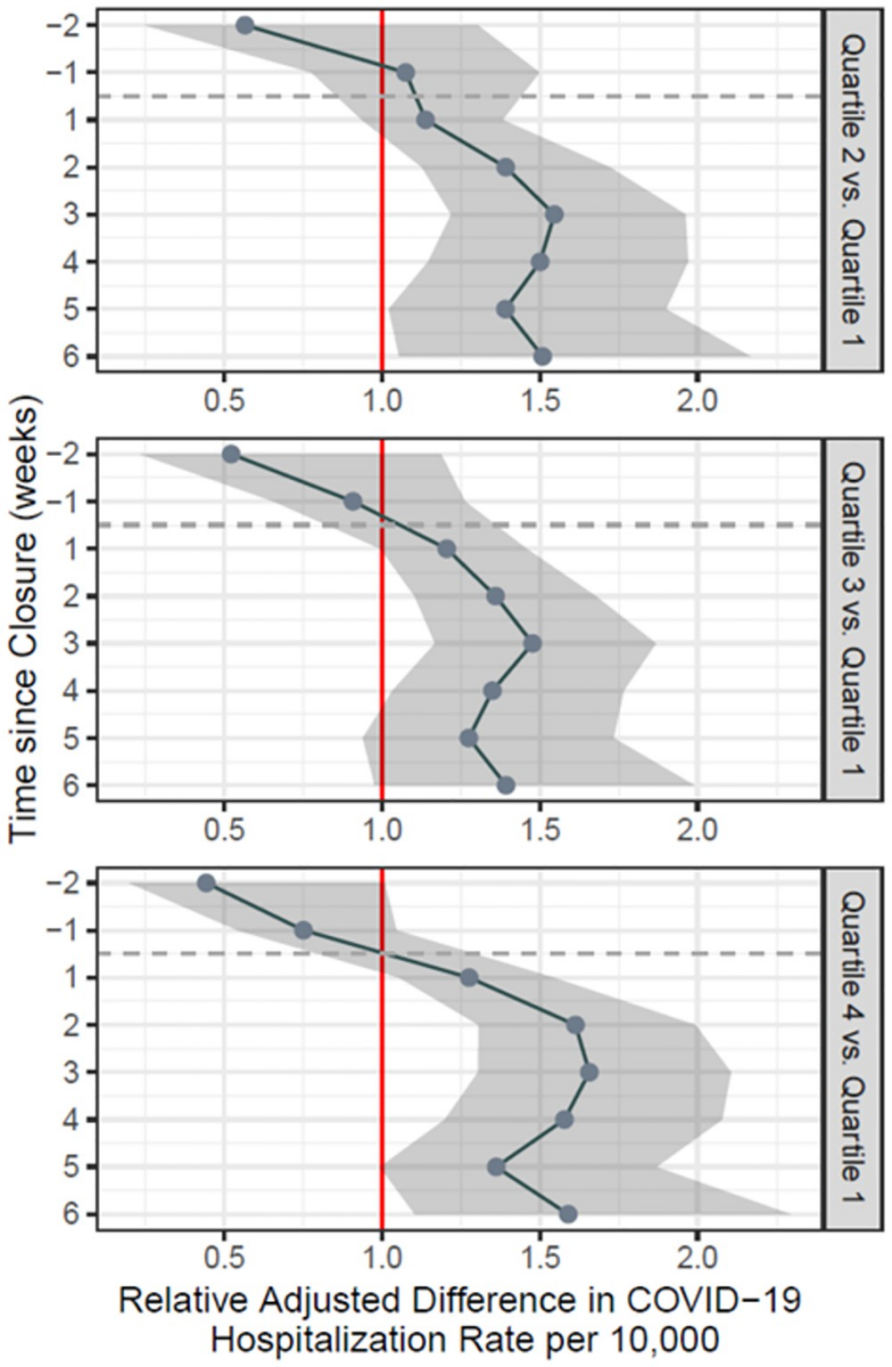
Adjusted difference-in-difference estimates of the association between school closure and COVID-19 hospitalization rates for patients older than 55 years, with ZCTAs grouped by quartiles of multigenerational housing proportions (with quartile 1 as reference). In all panels, the vertical axis represents the number of weeks relative to the school closure order, starting two weeks before the order and going up to six weeks afterwards. These estimates are adjusted for the following ZCTA-level covariates: percentage of overcrowded households (defined as estimated number of housing units with more than one occupant per room, divided by the number of occupied housing units), percentage of White residents, percentage of residents living under the Federal Poverty Line, and median income in 2018 USD–all taken from the American Community Survey 2018 5-year estimates.

**Fig 3:**
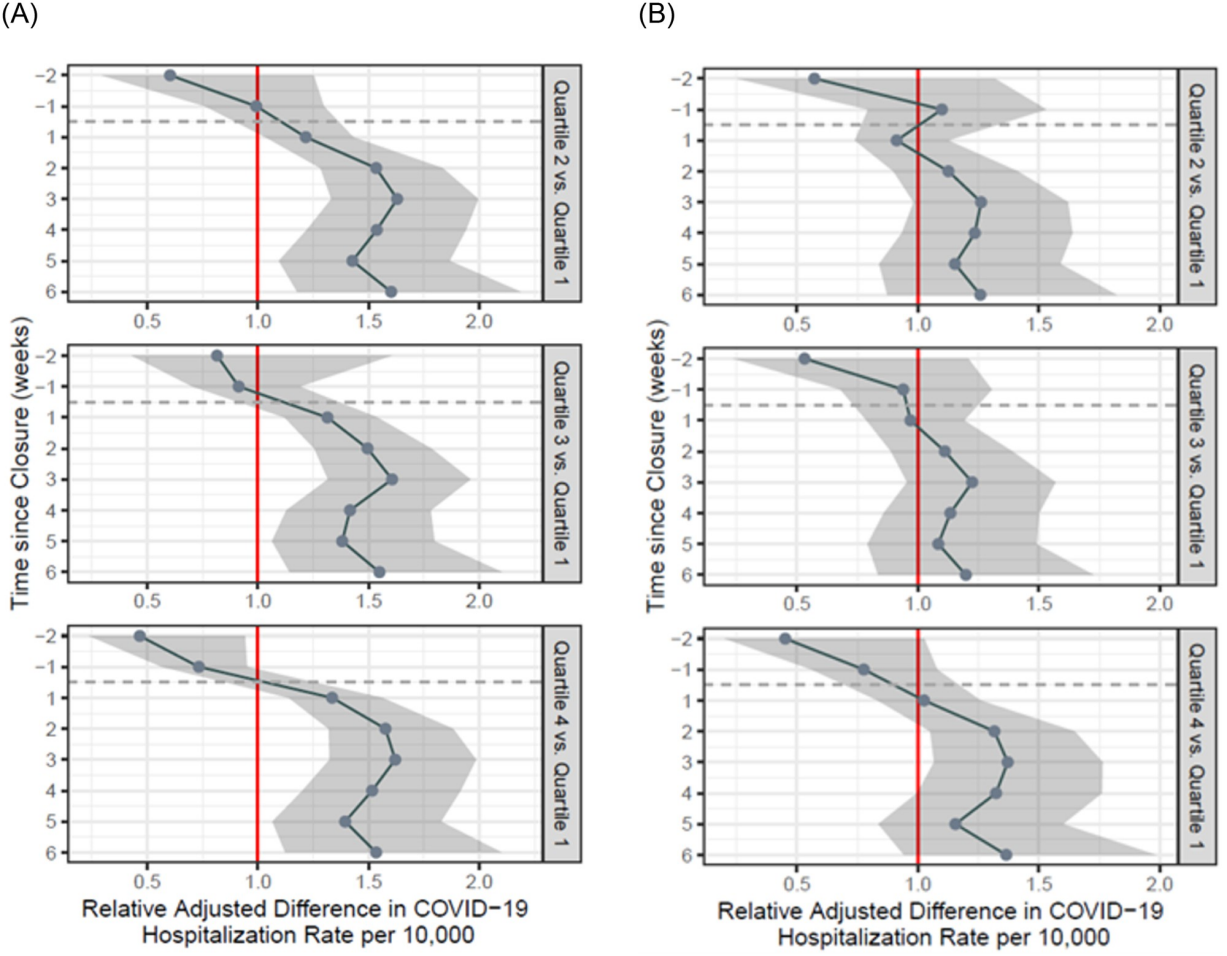
A. Adjusted difference-in-difference estimates of the association between school closure and COVID-19 hospitalization rates for all age groups, with ZCTAs grouped by quartiles of multigenerational housing proportions (with quartile 1 as reference). In all panels, the vertical axis represents the number of weeks relative to the school closure order, starting two weeks before the order and going up to six weeks afterwards. These estimates are adjusted for the following ZCTA-level covariates: percentage of overcrowded households, percentage of White residents, percentage of residents living under the Federal Poverty Line, and median income in 2018 USD–all taken from the American Community Survey 2018 5-year estimates. B. Patients over 55 years of age, with population estimates adjusted for pandemic-related flight. ZCTA-level population shifts were calculated from census tract estimates of population shifts given by publicly available cellular device data from Teralytics between January 1, 2020 and April 15, 2020, obtained from the New York Times.

Upon finding that there was significant spatial autocorrelation in our dependent variable (Moran’s I: 0.575, p = 0.001 –S1 Fig), our final model was a Bayesian version of a Poisson regression model that we fit using the integrated nested Laplace approximation (INLA) method. The INLA is a computationally efficient method for fitting models to data exhibiting spatial or temporal structure, and has been used in other ecological analyses of COVID-19 [10, 24]. The coefficients of our multivariable INLA model, as well as their 95% Bayesian credible intervals, are presented in Figs 2 and 3. These coefficients represent the percent increase in risk of COVID-19 hospitalization in ZCTAs with higher proportions of households that are multigenerational (quartiles 2–4) compared to ZCTAs with lower proportions (quartile 1).

### Secondary analyses

We performed two subsequent analyses. The first analysis modeled all COVID-19 hospitalizations, regardless of age group, using the same covariates in as the primary analysis.

The second analysis employed the same model as the primary analysis, but also accounted for population shifts related to pandemic-related flight. GPS data suggested that large segments of the population left NYC during the first COVID-19 wave, with residents from wealthier neighborhoods leaving in larger proportions [25]. We adjusted ZCTA-level population estimates according to census tract-level population shifts that were recorded in publicly available cellular device data from Teralytics between January 1, 2020 and April 15, 2020. This data was obtained from the New York Times [26]. Informed by different data from Descartes Labs [25], we modeled population shifts due to pandemic-related flight as time-varying, with a stable population estimate reached two weeks after lockdown.

### Sensitivity analyses

We conducted two sensitivity analyses to understand the robustness of our findings. To test whether our results could be explained by other potentially confounding socioeconomic characteristics, we expanded the number of ZCTA-level control covariates to include the proportion of essential workers who were more likely to work in the community and not at home (calculated according to methodology described in [23, 27]), as well as the prevalence of COVID-19 related clinical risk factors such as obesity, diabetes, coronary heart disease, smoking and chronic obstructive airway disease. Second, we included the proportion of overcrowded households (defined above) as an independent variable in our model along with quartiles of multigenerational households. This tested the robustness of the hypothesized relationship between the prevalence of multigenerational households and COVID-19 hospitalization rates after accounting for the impact of overcrowded ZCTAs during the city-wide lockdown. Because age-specific chronic disease prevalence statistics were not available at the ZCTA level, these sensitivity analyses were conducted using COVID-19 hospitalization data for all ages.

The study protocol was approved by Weill Cornell Medical College of Cornell University Institutional Review Board (IRB), protocol # 20–03021681, with consent given in writing. All analyses were conducted in GeoDa version 1.16, QGIS version 3.16.2, and R version 3.6.2. Data were analyzed between January 20 and May 25, 2021.

## Results

### Unadjusted estimates

Across 166 ZCTAs in NYC, there were 51,008 individuals during the study period who were hospitalized with COVID-19, 71.05% of whom were patients over 55 years. In moving from the first to the fourth quartile of multigenerational housing prevalence (i.e. from ZCTAs with the smallest proportions of multigenerational households to ZCTAs with the largest proportions), the total number of COVID-19 hospitalizations in each quartile increased overall, as well as per 10,000 population (Table 1). Compared to ZCTAs in the first quartile, the number of COVID-19 hospitalizations was more than two-fold higher in the fourth quartile (Q1: 33.95 hospitalizations per 10,000 vs. Q4: 82.21 hospitalizations per 10,000).

As ZCTAs contained more multigenerational households, the median income fell by more than half (Q1: $105,975.40 vs. Q4: $49,647.22), the proportion of White residents fell by more than a third (Q1: 72.49% vs. Q4: 20.68%), the proportion of residents below the federal poverty line approximately doubled (Q1: 11.01% vs. Q4: 25.62%), and the proportion of those residing in overcrowded housing increased (Q1: 4.92% vs. Q4: 13.53%).

Fig 1 displays the unadjusted daily trend of COVID-19 hospitalizations in ZCTAs in each quartile of multigenerational households. The peak of COVID-19 hospitalizations in NYC took place on March 30, 2020, after which hospitalizations trended downwards daily. At the peak of hospitalizations, there is almost a three-fold difference in unadjusted COVID-19 hospitalization counts between quartiles (March 30, 2020; Q1: 1.28 cases per 10,000 vs. Q4: 3.16 cases per 10,000).

### Adjusted estimates

For ZCTAs in each quartile of multigenerational housing prevalence, Fig 2 shows estimates of the adjusted relative differences in COVID-19 hospitalization rates for patients over 55 years of age, compared to quartile 1 of ZCTAs, during the eight-week study period. Prior to the lockdown, there were no significant differences in COVID-19 hospitalization rates relative to quartile 1 (i.e., ZCTAs with the lowest proportion of multigenerational households). After the lockdown began, there was a consistent and significant increase in COVID-19 hospitalization rates across all quartiles of ZCTAs. This difference persists over the study period for all quartiles. The greatest difference in adjusted COVID-19 hospitalization rates was noted three weeks after lockdown: Q2 vs. Q1: 1.55 (95% CI: 1.22–1.96); Q3 vs. Q1: 1.48, (95% CI: 1.17–1.87); Q4 vs. Q1: 1.66, (95% CI: 1.30–2.10). These findings suggest that at three weeks after lockdown, the risk of hospitalization from COVID-19 in ZCTAs with higher proportions of multigenerational households was 55–66% greater than in ZCTAs with the lowest proportions of multigenerational households.

### Secondary analysis

Secondary analyses of all patients hospitalized with COVID-19 demonstrated similar findings to the primary analysis (Fig 3A). Relative differences between adjusted COVID-19 hospitalization rates in the first quartile and second, third, and fourth quartiles were greatest at week three: Q2 vs. Q1: 1.62 (95% CI: 1.33–2.00); Q3 vs. Q1: 1.60, (95% CI: 1.32–1.96); Q4 vs. Q1: 1.62, (95% CI: 1.32–1.99). However, the pre-closure adjusted COVID-19 hospitalization rate was higher in the first quartile compared to the fourth quartile, and the difference was statistically significant.

Fig 3B shows results that account for pandemic-related flight. Throughout the study period, the differences in adjusted COVID-19 hospitalization rates were not statistically significant between Q1 and Q2 as well as Q1 and Q3. However, the relative difference in COVID-19 hospitalization rates between Q4 and Q1 increases to a statistically significant difference of 37% (95% CI: 1.07–1.76) three weeks after lockdown.

### Sensitivity analysis

Estimates from the sensitivity analyses were largely consistent with those from the primary analysis (S2 Fig). When we considered multigenerational households and overcrowded households together in the same modeling strategy, we found that the association between city-wide lockdown, ZCTAs with higher proportions of multigenerational households, and COVID-19 hospitalization rates was attenuated, but remained significant (S3 Fig).

## Discussion

This first empirical study examines the relationship between city-wide lockdown and COVID-19 hospitalization rates amongst older adults in multigenerational households in NYC. We found that the lockdown was associated with a transient and disproportionate increase in hospitalizations among individuals older than 55 years residing in ZCTAs with higher proportions of multigenerational households, after adjusting for socioeconomic characteristics. The estimated association was consistent with the known transmission dynamics in multigenerational households during the exponential growth phase of COVID-19 in NYC, and had its greatest effect three weeks after lockdown measures were implemented. These results were attenuated by pandemic-related flight, but remained significant in the ZCTAs with the highest proportions of multigenerational households.

Our results add crucial ecological evidence to the emerging literature on household structure and COVID-19 risk. Moreover, they add empirical evidence to simulation studies that postulated deleterious effects of school closures, which were the first city-wide lockdown measure introduced [18]. By assessing the effect of a city-wide lockdown with temporal and geographic specificity, our study contributes to this body of literature by illustrating how such blanket public health measures may contribute to uneven and transiently increased COVID-19 risk among older individuals residing in multigenerational households. Multigenerational households follow a socioeconomic gradient [28], and racial/ethnic differences between single and multi-generational households exist [29, 30]. The well-documented racial and ethnic disparities in COVID-19 related outcomes have been explained through mechanisms such as the disproportionate burden of disease among minority groups [31, 32], limited economic opportunity [33], and other structural determinants [34]. This study adds another critical element to this discussion by introducing evidence for a link between multigenerational household structure and COVID-19 hospitalization. Our findings are in accord with various biological mechanisms of COVID-19 transmission, such the relative differences in age-related outcomes from COVID-19, the role of proximity in viral transmission [2] and subsequent severity of infection [4], and the 14-day incubation period (evidenced by the greatest difference in hospitalization rates being seen three weeks after lockdown measures).

Our findings should not be interpreted to suggest that NPIs, such as city-wide lockdowns, do not work. They clearly do. Instead, they suggest that the impact of such measures is not uniform across households and neighborhoods. Early reports in the pandemic underlined the age-dependent morbidity related to SARS-CoV2 infection [35]. Therefore, it is logical that mandated home confinement including of those including school-age children during COVID-19’s exponential rise may have disproportionately affected households where both younger and older individuals reside [36]. Our analysis does not establish causality, but it does suggest that NYC’s experience with lockdown during the initial COVID-19 wave reflects this phenomenon. Identifying whether these findings are replicable in other cities, as well as assessing other potential secondary effects from different social distancing measures, remains an important area of future research.

### Limitations

This study has five limitations. First, despite attempts to address unobservable confounding influences, we cannot rule out the role of residual confounding due to the observational nature of the study. This is particularly relevant in the use of control covariates which acted as proxies for socioeconomic risk at the ZCTA level. Although we were careful to note which factors may act to mediate and confound the relationship under study, there could be other unmeasured time-varying factors. Second, because our data was collected at the ZCTA level, we were not able to evaluate individual COVID-19 hospitalization risk. Third, our analysis does not quantify the relative hospitalization risk in multigenerational households incurred by not undertaking city-wide lockdown; this risk is likely to outweigh the impact of lockdown. Nonetheless, our results indicate that the risks and benefits of lockdown vary across different populations, with particularly stark consequences for multigenerational households. Fourth, hospitalization rates may not accurately reflect the remaining population base of ZCTAs in NYC because of pandemic-related flight, which favored wealthier neighborhoods [25]. This may have reduced the total population at risk in our analysis, particularly in ZCTAs with lower proportions of multigenerational households, and led to overestimation of the neighborhood-level effects described. However, our use of mobile phone data attempted to address this issue. Lastly, we could not test the parallel trends assumption of our difference-in-difference design. However, pre-lockdown differences in adjusted COVID-19 hospitalization rates between quartiles were insignificant.

## Conclusions

In its first COVID-19 wave, NYC’s lockdown was associated with increases in COVID-19 hospitalization rates for individuals over 55 years of age in ZCTAs with higher proportions of multigenerational households. These findings highlight unanticipated interactions between structural factors such as housing and social distancing measures that may have contributed to greater transmission risks for select vulnerable groups in the pandemic.

## Supporting information

S1 Fig Map of New York City with cumulative COVID-19 hospitalizations by ZIP code tabulation area from February 23 to May 23, 2020. (DOCX)

S2 Fig Adjusted difference-in-difference estimates of the association between school closure and COVID-19 hospitalization rates for all age groups, with ZCTAs grouped by quartiles of multigenerational housing proportions (with quartile 1 as reference) and all socioeconomic and clinical risk factors included as covariates. This model controlled for the following covariates: ZCTA-level prevalence, as a percentage, of: adults who are obese (defined as body mass index [BMI] ≥ 30 kg/m^2^), adults who smoke, and adults with coronary heart disease, hypertension, diabetes, asthma, or chronic obstructive pulmonary disease (COPD)–taken from the CDC 500 Cities Dataset; additional socioeconomic factors: ZCTA-level estimates of total population, percentage of residents living below the federal poverty line (FPL), median income in 2018 USD, percentage of White residents, and percentage of overcrowded households (defined as estimated number of housing units with more than one occupant per room, divided by the number of occupied housing units)—all taken from the ACS 5-year estimates 2018; and percentage of essential workers by ZCTA, identified from service-oriented non-public roles using Census Industrial Classification Codes in the following categories: 1) public transit workers, 2) grocery, convenience and drug store workers, 3) trucking, warehouse and postal service workers, 4) healthcare workers, 5) childcare, homeless, food and family service workers, and 6) building cleaning service workers–we replicated the same methodology employed by the New York City Office of the Comptroller (Scott S [2020] New York City’s Frontline Workers. New York City: City of New York, Office of the Comptroller).
(DOCX)

S3 Fig A. Adjusted difference-in-difference estimates of the association between school closure and COVID-19 hospitalization rates for all age groups, with ZCTAs grouped by quartiles of multigenerational housing proportions (with quartile 1 as reference), and with quartiles of overcrowded ZCTAs also included as a covariate. This model controlled for the following covariates: percentage of residents living below the federal poverty line (FPL), median income in 2018 USD, percentage of White residents, percentage of overcrowded households (defined as estimated number of housing units with more than one occupant per room, divided by the number of occupied housing units)—all taken from the ACS 5-year estimates 2018. B. Adjusted difference-in-difference estimates of the association between school closure and COVID-19 hospitalization rates for all age groups, with ZCTAs grouped by quartiles of multigenerational housing proportions (with quartile 1 as reference), and with quartiles of overcrowded ZCTAs also included as a covariate. This model controlled for the same covariates as described in S3A Fig. This plot presents the coefficients and 95% Bayesian credible intervals of the interaction between time in weeks (indexed at *t* = 0 for the week of school closure) and the quartile of overcrowded housing proportions that each ZCTA belongs to, after accounting for the interaction effect between time and the ZCTA’s quartile of multigenerational housing.
(DOCX)

S1 File Model specification for primary analysis. (DOCX)

S1 Data (XLSX)
