# Supplementary material for: Association between city-wide lockdown and COVID-19 hospitalization rates in multigenerational households in New York City
Source: PLoS One. 2022 Mar 30;17(3):e0266127. doi: 10.1371/journal.pone.0266127 (PMC8967012; doi:10.1371/journal.pone.0266127)

**S1 Fig. Map of New York City with cumulative COVID-19 hospitalizations by ZIP Code Tabulation Area from February 23 to May 23, 2020**


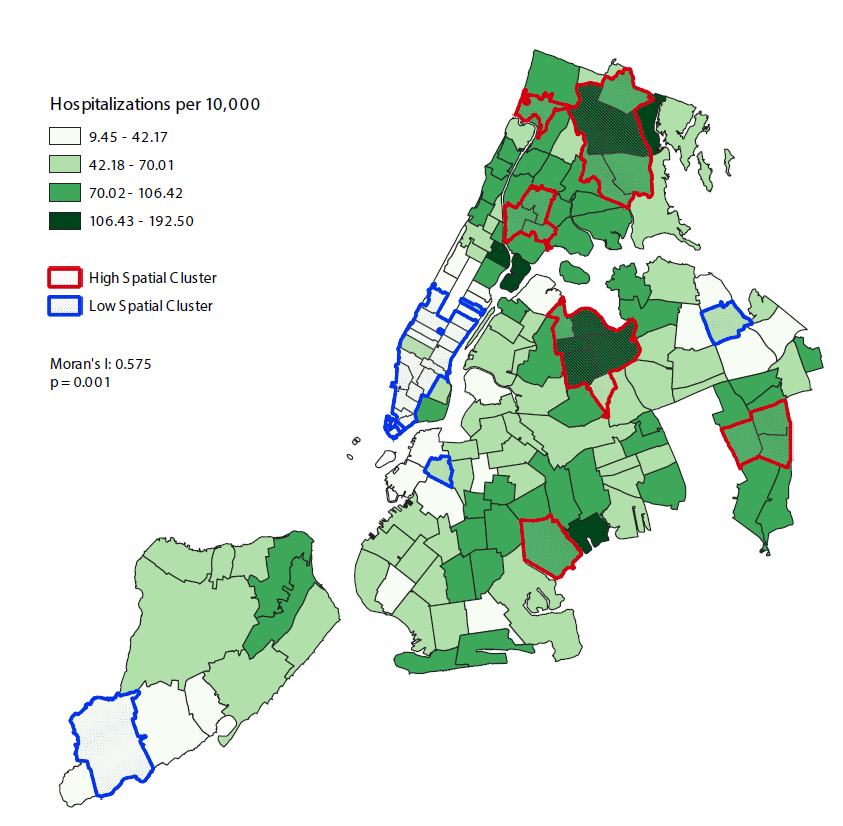

Supplement: S1 Fig — (DOCX) [file pone.0266127.s001.docx]
