# Supplementary material for: Association between city-wide lockdown and COVID-19 hospitalization rates in multigenerational households in New York City
Source: PLoS One. 2022 Mar 30;17(3):e0266127. doi: 10.1371/journal.pone.0266127 (PMC8967012; doi:10.1371/journal.pone.0266127)
Supplement: S2 Fig — This model controlled for the following covariates: ZCTA-level prevalence, as a percentage, of: adults who are obese (defined as body mass index [BMI] ≥ 30 kg/m2), adults who smoke, and adults with coronary heart disease, hypertension, diabetes, asthma, or chronic obstructive pulmonary disease (COPD)–taken from the CDC 500 Cities Dataset; additional socioeconomic factors: ZCTA-level estimates of total population, percentage of residents living below the federal poverty line (FPL), median income in 2018 USD, percentage of White residents, and percentage of overcrowded households (defined as estimated number of housing units with more than one occupant per room, divided by the number of occupied housing units)—all taken from the ACS 5-year estimates 2018; and percentage of essential workers by ZCTA, identified from service-oriented non-public roles using Census Industrial Classification Codes in the following categories: 1) public transit workers, 2) grocery, convenience and drug store workers, 3) trucking, warehouse and postal service workers, 4) healthcare workers, 5) childcare, homeless, food and family service workers, and 6) building cleaning service workers–we replicated the same methodology employed by the New York City Office of the Comptroller (Scott S [2020] New York City’s Frontline Workers. New York City: City of New York, Office of the Comptroller). (DOCX) [file pone.0266127.s002.docx]

**S2 Fig. Difference-in-Differences Estimates of the Association Between School Closure and Adjusted COVID-19 Hospitalization Rates by Quartiles of Multigenerational ZIP Codes with Inclusion of all Socioeconomic and Clinical Risk Factors,^1^ with Quartile 1 as Reference^2^**


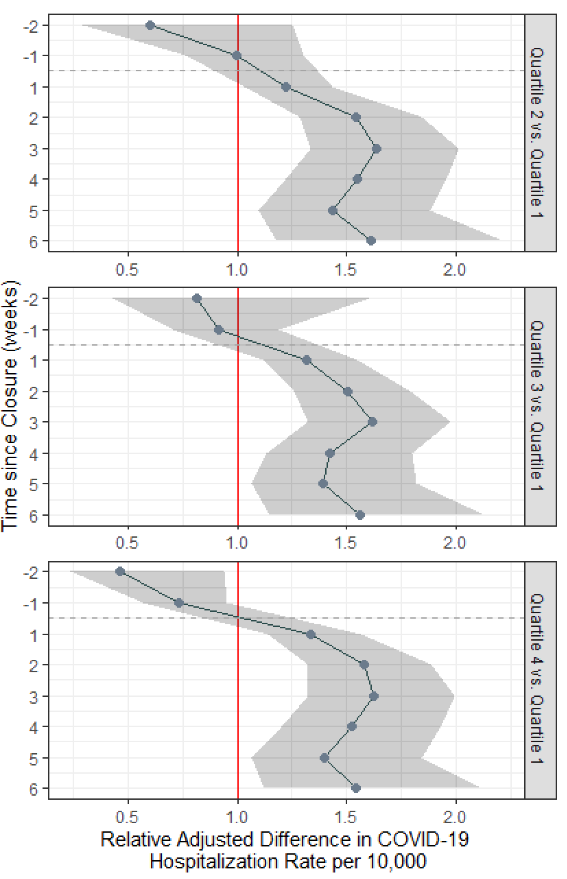


1 This model controlled for the following covariates: ZCTA-level prevalence (in percentage) of obese adults (defined as body mass index [BMI] ≥ 30 kg/m2), adults who smoke, and adults with coronary heart disease, hypertension, diabetes, asthma, or chronic obstructive pulmonary disease (COPD) – taken from the CDC 500 Cities Dataset; and additional socioeconomic factors: ZCTA-level estimates of total population, percentage of patients living below the federal poverty line (FPL), median income , percentage of White residents, and percentage of overcrowded households (defined as estimated number of housing units with more than one occupant per room, divided by the number of occupied housing units) - all taken from the ACS 5-year estimates 2018 - , and percentage of essential workers by ZCTA, identified from service-oriented non-public roles using Census Industrial Classification Codes in the following categories: 1) public transit workers; 2) grocery, convenience and drug store workers; 3) trucking, warehouse and postal service workers; 4) healthcare workers; 5) childcare, homeless, food and family service workers; and 6) building cleaning service workers – we replicated the same methodology as employed by the New York City Office of the Comptroller (Scott S [2020] New York City's Frontline Workers. New York City: City of New York, Office of the Comptroller).

2 All COVID-19 hospitalizations included in this analysis
