# Supplementary material for: Association between city-wide lockdown and COVID-19 hospitalization rates in multigenerational households in New York City
Source: PLoS One. 2022 Mar 30;17(3):e0266127. doi: 10.1371/journal.pone.0266127 (PMC8967012; doi:10.1371/journal.pone.0266127)
Supplement: S3 Fig — A. Adjusted difference-in-difference estimates of the association between school closure and COVID-19 hospitalization rates for all age groups, with ZCTAs grouped by quartiles of multigenerational housing proportions (with quartile 1 as reference), and with quartiles of overcrowded ZCTAs also included as a covariate. This model controlled for the following covariates: percentage of residents living below the federal poverty line (FPL), median income in 2018 USD, percentage of White residents, percentage of overcrowded households (defined as estimated number of housing units with more than one occupant per room, divided by the number of occupied housing units)—all taken from the ACS 5-year estimates 2018. B. Adjusted difference-in-difference estimates of the association between school closure and COVID-19 hospitalization rates for all age groups, with ZCTAs grouped by quartiles of multigenerational housing proportions (with quartile 1 as reference), and with quartiles of overcrowded ZCTAs also included as a covariate. This model controlled for the same covariates as described in S3A Fig. This plot presents the coefficients and 95% Bayesian credible intervals of the interaction between time in weeks (indexed at t = 0 for the week of school closure) and the quartile of overcrowded housing proportions that each ZCTA belongs to, after accounting for the interaction effect between time and the ZCTA’s quartile of multigenerational housing. (DOCX) [file pone.0266127.s003.docx]

**S3 Fig. Difference-in-Differences Estimates of the Association Between School Closure and Adjusted COVID-19 Hospitalization Rates by Quartiles of Multigenerational ZIP Codes with the inclusion of Quartiles of Overcrowded ZIP codes^1^, with Quartile 1 as reference^2^**

**a) Quartiles of Multigenerational ZIP codes**


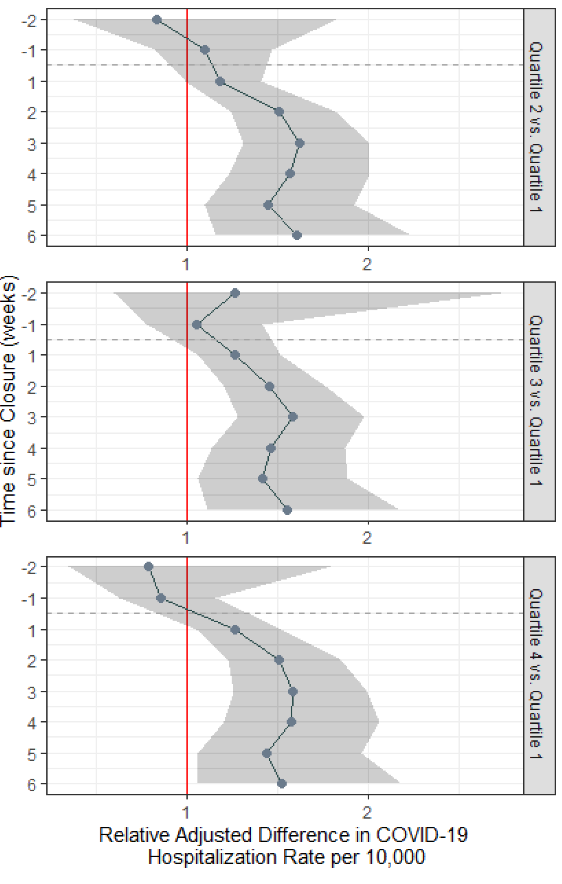


**3b) Quartiles of Overcrowded ZIP codes^3^**


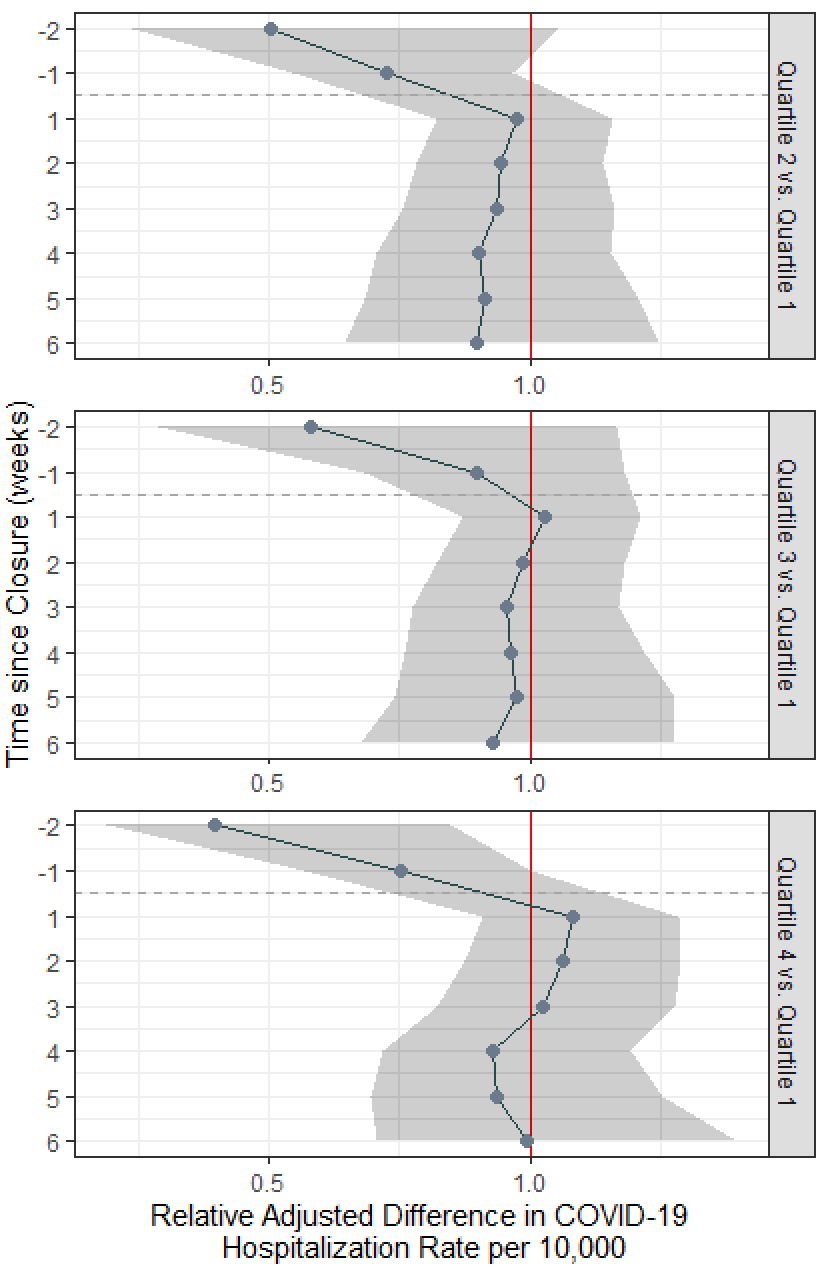


1 Models in A) and B) controlled for the following covariates: percentage of patients living below the federal poverty line (FPL), median income in 2018 USD, percentage of overcrowded households (defined as estimated number of housing units with more than one occupant per room, divided by the number of occupied housing units) and percentage of White residents (all taken from the ACS 5-year estimates 2018

2 All COVID-19 hospitalizations included in this analysis

3 Represents the coefficients and 95% Bayesian credible intervals of the interaction between time in weeks (indexed at t = 0 for the week of school closure) and quartiles of ZCTAs with overcrowded households, after accounting for the interaction effect between time and quartiles of ZCTAs with multigenerational households.
