# Supplementary material for: Association between city-wide lockdown and COVID-19 hospitalization rates in multigenerational households in New York City
Source: PLoS One. 2022 Mar 30;17(3):e0266127. doi: 10.1371/journal.pone.0266127 (PMC8967012; doi:10.1371/journal.pone.0266127)
Supplement: S1 File — (DOCX) [file pone.0266127.s004.docx]

**S1 File. Model specification for primary analysis**

The generalized linear model we employed for the mean hospitalization count in each ZCTA in each week is of the following form:

$$E\left( \log\left( 10,000\times\frac{Y_{it}}{\mathrm{Populatio}n_{i}} \right) \right)=\beta_{0}+\sum_{j=-2}^{6} \beta_{j, MG_{i}}\left( \mathrm{Tim}e_{j}\times MG_{i} \right)+\mu_{i}+\nu_{i}+\theta_{t}+\varepsilon_{ijt}$$

where:

- $Y_{it}$ denotes the number of hospitalizations in ZCTA $i$in week $t$, where $t$ ranges from -2 (two weeks before school closure) to 6 (six weeks after school closure). The date of school closure is March 16, 2020. The $t$ = -2 count aggregates hospitalizations from the weeks of February 23, 2020 and March 1, 2020.
- $\mathrm{Tim}e_{j}$ is a binary variable indicating whether or not $j = t$. Time $t=0$ (the week of lockdown and school closures) is treated as the reference level.
- $MG_{i}$is a categorical variable indicating which quartile of multigenerational housing ZCTA falls into (Q1, Q2, Q3, Q4). Quartile 1 is treated as the reference level.
- The quartile-specific coefficients $\beta_{j, MG_{i}}$ are plotted in Figures 2 and 3. These coefficients can be interpreted as the difference in log hospitalization counts between quartile $MG_{i}$and quartile 1 at time $j$, relative to time 0. When exponentiated, they are interpreted as multiplicative factors rather than differences.
- $\mu_{i}$ denotes ZCTA-level fixed effects, including the effect of the ZCTA’s median income, percentage of residents who are white, and percentage of residents below the federal poverty threshold.
- $\nu_{i}$ denotes a spatial random effect term for ZCTA $i$. In particular, it is a conditionally autoregressive term with a Besag-York-Mollié specification; this means that conditioned on the spatial random effect terms for all other ZCTAs, $\nu_{i}$ is modeled as normally-distributed with mean $\frac{1}{\left| N_{i} \right|}\sum_{j\in N_{i}} \nu_{j}$, where $N_{i}$ are the indices of the immediately-neighboring ZCTAs.
- $\theta_{t}$ denotes a weekly time fixed effect that captures trends in the outcome not explained by the other terms in the model.
- $\mathrm{Populatio}n_{i}$ is the population of ZCTA $i$.
